# Use of automated quantitative pupillometric evaluation for monitoring the severity of diabetic retinopathy

**DOI:** 10.5935/0004-2749.20210007

**Published:** 2025-02-02

**Authors:** Veysel Cankurtaran, Cagri Ilhan, Kemal Tekin, Mehmet Citirik, Emre Dirican, Eren Gurkan

**Affiliations:** 1 Ophthalmology Department, Mustafa Kemal University, Hatay, Turkey; 2 Ophthalmology Department, Hatay State Hospital, Hatay, Turkey; 3 Ophthalmology Department, Ercis State Hospital, Van, Turkey; 4 Ankara Ulucanlar Eye Education and Research Hospital, University of Health Sciences, Ankara, Turkey; 5 Biostatistics Department, Mustafa Kemal University, Hatay, Turkey; 6 Endocrinology Department, Mustafa Kemal University, Hatay, Turkey

**Keywords:** Diabetic retinopathy, Diabetes mellitus, Diagnostic techniques, ophthalmological, Pupil, Reflex, pupillary, Retinopatia diabética, Diabetes Mellitus, Técnicas de diagnóstico oftalmológico, Pupila, Reflexo pupilar

## Abstract

**Purpose:**

We aimed to evaluate the use of automated quantitative static and dynamic pupillometry in screening patients with type 2 diabetes mellitus and different stages of diabetic retinopathy.

**Method:**

155 patients with type 2 diabetes mellitus (diabetes mellitus group) were included in this study and another 145 ageand sex-matched healthy individuals to serve as the control group. The diabetes mellitus group was divided into three subgroups: diabetes mellitus without diabetic retinopathy (No-diabetic retinopathy), nonproliferative diabetic retinopathy, and proliferative diabetic retinopathy. Static and dynamic pupillometry were performed using a rotating Scheimpflug camera with a topography-based system.

**Results:**

In terms of pupil diameter in both static and dynamic pupillometry (p<0.05), statistically significant differences were observed between the diabetes mellitus and control groups and also between the subgroups No-diabetic retinopathy, nonproliferative diabetic retinopathy, and proliferative diabetic retinopathy subgroups. But it was noted that No-diabetic retinopathy and nonproliferative diabetic retinopathy groups have showed similarities in the findings derived from static pupillometry under mesopic and photopic conditions. The two groups also appeared similar at all points during the dynamic pupillometry (p>0.05). However, it could be concluded that the proliferative diabetic retinopathy group was significantly different from the rest of the subgroups, No-diabetic retinopathy and nonproliferative diabetic retinopathy groups, in terms of all the static pupillometry measurements (p<0.05). The average speed of dilation was also significantly different between the diabetes mellitus and control groups and among the diabetes mellitus subgroups (p<0.001). While weak to moderate significant correlations were found between all pupil diameters in static and dynamic pupillometry with the duration of diabetes mellitus (p<0.05 for all), the HbA1c values showed no statistically significant correlations with any of the investigated static and dynamic pupil diameters (p>0.05 for all).

**Conclusion:**

This study revealed that the measurements derived from automated pupillometry are altered in patients with type 2 diabetes mellitus. The presence of nonproliferative diabetic retinopathy does not have a negative effect on pupillometry findings, but with proliferative diabetic retinopathy, significant alterations were observed. These results suggest that using automated quantitative pupillometry may be useful in verifying the severity of diabetic retinopathy.

## Abstract (pt)

**Objetivos:**

Procuramos avaliar o uso da pupilo metria estática e dinâmica quantitativa automatizada na triagem de pacientes com *diabetes mellitus* tipo 2 e em diferentes estágios de retinopatia diabética.

**Métodos:**

Cento e cinquenta e cinco pacientes com *diabetes mellitus* tipo 2 (grupo com *diabetes mellitus*) foram incluídos neste estudo e outros 145 controles saudáveis pareados por idade e sexo para server como grupo controle. O grupo com *diabetes mellitus* foi dividido em três subgrupos: *diabetes mellitus* sem retinopatia diabética (retinopatia não diabética), retinopatia diabética não proliferativa e retinopatia diabética proliferativa. A pupilometria estática e dinâmica foi realizada utilizando uma camera rotative Scheimpflug com um sistema baseado em topografia.

**Resultados:**

Em termos de diâmetro da pupila, tanto na pupilometria estática quanto na dinâmica (p<0,05), foram observadas diferenças estatisticamente significantes entre os grupos *diabetes mellitus* e controle e também entre os subgrupos retinopatia não diabética, retinopatia diabética não proliferativa e retinopatia diabética proliferativa. Mas foi observado que os grupos de retinopatia não diabética e retinopatia diabética não proliferativa mostraram semelhanças nos achados derivados da pupilometria estática em condições mesópicas e fotópicas. Os dois grupos também pareciam semelhantes em todos os pontos durante a pupilometria dinâmica (p>0,05). No entanto, pode-se concluir que o grupo de retinopatia diabética proliferative foi sugnificativamente diferente do restante dos subgrupos, retinopatia não diabética e retinopatia diabética não proliferativa, em termos de todas as medidas de pupilometris estática (p<0,05). A velocidade média de dilatação também foi significativamente diferente entre os grupos *diabetes mellitus* e controle, e entre os subgrupos *diabetes mellitus* (p<0,001). Enquanto correlações significativas fracas a moderadas foram encontradas entre todos os diâmetros da pupila na pupilometria estática e dinâmica com a duração do *diabetes mellitus* (p<0,05 para todos), os valores de HbA1c não mostraram correlações estatisticamente significantes com nenhum dos diâmetros da pupila estática e dinâmica investigados (p>0,05 para todos).

**Conclusão:**

Este estudo revelou que as medidas derivadas da pupilometria automatizada estão alteradas em pacientes com *diabetes mellitus* tipo 2. A presença de retinopatia diabética não proliferativa não afeta negativamente os achados pupilométricos, mas com a retinopatia diabética proliferative, alterações significativas foram observadas. Estes resultados sugerem que o uso da pupilometria quantitativa automatizada pode ser útil na verificação gravidade da retinopatia diabética.

## INTRODUCTION

The size and function of the pupils are directly controlled by the autonomic nervous system through the sphincter (circular) and dilatator (radial) muscles of the iris. The parasympathetic neuronal axons, originating from the Edinger-Westphal nucleus, synapse on the ciliary ganglion and innervate the sphincter muscle of the pupil. At the same time, the dilatator muscle of the pupil is innervated by sympathetic neuronal axons originating from the posterolateral hypothalamus that synapse on the intermediolateral cell column of C8 to T2 and the superior cervical ganglion. These muscles and nerves work in coordination, providing optimal retinal lightning and perfect depth of focus via optimal pupil size^(1)^.

Diabetic retinopathy (DR), diabetic macular edema, and neovascular glaucoma are well-known ocular complications of diabetes mellitus (DM), but all the layers of the eye globe, from the precorneal tear film to the lamina cribrosa, are vulnerable to experiencing more manifestations of DM, which could lead to more complications than just these^(2,3)^. Diabetic autonomic neuropathy (DAN) is another common ophthalmological complication, but it is less studied and less understood than the aforementioned three. Smaller resting pupil diameter and reflex amplitudes are relatively well-recognized as early clinical manifestations of DAN, but pupil diameter in static pupillometry under scotopic, mesopic, and photopic conditions and dilation capacity and speed have not been described extensively in different stages of DR^(4,5)^.

Examining the pupillomotor function is a useful method for screening for DAN, which can be incorporated in a wide range of techniques from simple scale measurements to infrared observation^(6)^. Although the best way to measure the pupil size has not been definitively determined, automated quantitative pupillometry is considered as the best modern method for improving the screening for autonomic dysfunction^(7)^. However, despite its objective, repeatable, and quantitative measurements on the pupillomotor function, automated pupillometry requires specific equipment, trained operators, and active patient participation.

In this study, we sought to evaluate the findings of automated quantitative static and dynamic pupillometry in type 2 DM patients with different stages of DR.

## METHODS

This prospective study was carried out at an ophthalmology clinic of a university hospital, with approval granted by the local research ethics committee. The aims and methods of the study were explained to the selected participants in detail, and informed consent was obtained thereafter for each subject. All procedures were performed in accordance with the ethical standards of the Declaration of Helsinki for human subjects.

### Study subjects

In all eligible study participants, DM was previously detected by the corresponding internal medicine department. The status of DR was assessed by fundus photography and confirmed with fluorescein angiography and optical coherence tomography. Early Treatment of Diabetic Retinopathy Study criteria were utilized to define various stages of DR. Selected ageand sex-matched healthy controls (control group) had visited the ophthalmology clinic for a routine ocular examination and/or presbyopic complaints. Cases with any systemic disease in the control group were excluded from this study.

All subjects underwent detailed medical questioning and ophthalmological evaluation including manifest refraction, best-corrected visual acuity (BCVA) (all subjects had a 0.4 decimal or better BCVA finding with the Snellen chart), color vision, intraocular pressure measurement, slit-lamb biomicroscopy, and dilated fundus examination. Colored fundus photography, fundus fluorescein angiography, and/or optical coherence tomography were performed for the DM group by the same clinician (V. C.). The DM group was divided into three subgroups as follows: DM without DR (No-DR), nonproliferative DR (NPDR), and proliferative DR (PDR).^(8)^ Moreover, the duration of DM and the glycosylated hemoglobin (HbA1c) values were recorded for the patients with DM.

However, we excluded individuals who had a history of ocular trauma, glaucoma, uveitis, hyperopia, myopia or astigmatism of more than 1.00 diopters (D), herpetic corneal diseases, iris, or pupil anomalies, pseudoexfoliation, grades 3 or 4 cataract, retinal diseases that may affect the pupil, optic neuropathies, color vision deficiencies, and use of chronic topical ophthalmic medications. Subjects with other systemic diseases, especially affecting the central nervous system or urinary system and/or who were using systemic medications, were also excluded. Any patient with proliferative retinopathy associated with systemic diseases or localized retinal vascular and/or ocular inflammatory diseases was excluded as well. For the DM subjects, additional exclusion criteria included those who have undergone panretinal laser photocoagulation at any time or focal laser photocoagulation or intravitreal injection in the last year, respectively.

### Pupillometry

Automated pupillometry was performed by the same experienced clinician (V. C.) using a Sirius 3D Rotating Scheimpflug camera topography system with the software suite Phoenix v2.1 (Costruzione Strumenti Oftalmici, Scandicci, Italy). The examination was conducted in a completely dark room following dark adaptation for 20 minutes, and the measurements were obtained during the same hours each day (between 13:00 and 15:00 hours) to minimize the impact of circadian variation on pu pillary response^(9,10)^.

Static and dynamic pupillometry were evaluated under different illumination conditions. Static pupillometry was applied in three stages as follows: (1) scotopic measurement, in which the only visible light source was a light-emitting diode (LED) at 0.4 lux; (2) mesopic measurement, in which the disk was illuminated to bring the ambient light intensity to 4.0 lux; and (3) photopic measurement, in which the disc was illuminated to bring ambient intensity to 40.0 lux. The LED output had the following characteristics at T_A_ (ambient temperature) of 25°C: peak wavelength 660 nm, dominant wavelength 640 nm, spectral line half width 20 nm, capacitance 95 pF, forward voltage 1.85 V (typical), 2.5 V (maximum), and reverse current maximum of 10 µA. To prevent accommodative response, the subjects were advised to look straight ahead rather than at the LED source. The measurements of static and dynamic pupillometry were performed with capture started with the ring disc fully illuminated with 500 lux; the illumination was then switched off when capture started. Hereby, it could be possible to monitor dilation in conditions from photopic to scotopic and to evaluate the pupil diameter and offset instant by instant. After the measurements of dynamic pupillometry, the speed of change in pupil diameter was calculated using this formulation: V_average_ = ([Φ_t_ - Φ_t0_]/t); according to this formulation, average speed (mm/s) is equal to the difference in the pupil diameter (mm) between time (seconds) at sampling and at t = 0 divided by duration (seconds) between time at sampling and at t = 0^(10,11)^.

### Statistical analysis

The data of the study were analyzed using the Statistical Package for the Social Sciences version 24.0 for Windows software program (IBM Corp., Armonk, NY, USA). The data taken after examining the right eyes of the study subjects were subjected to statistical analysis. Descriptive data were presented as means ± standard deviations, minimums, and maximums, and the chi-square test was used to analyze these categorical variables. Normal distribution of the variables was checked by Kolmogorov-Smirnov test. Mahalanobis distance was reviewed for the variables that did not fit normal distribution, and then one-way analysis of variance and Student’s parametric t-tests were used. Post hoc tests (Tukey’s honestly significant difference) for pairwise comparisons were also performed. Meanwhile, Pearson correlation tests were used to investigate the correlations of pupil diameter with the duration of the DM and the HbA1c level. Statistically significance was set at p <0.05.

## RESULTS

This study included 155 subjects in DM group and 145 ageand sex-matched subjects in the control group. Demographic characteristics of the two groups are summarized in table 1. There were 49 patients in the No-DR subgroup, 53 patients in the NPDR subgroup, and 53 patients in the PDR subgroup, respectively. No statistically significant differences in age or gender were noted among these subgroups (p>0.05 for all). The mean durations of DM were 8.26 ± 3.96 years in the No-DR subgroup, 14.05 ± 4.75 years in the NPDR subgroup, and 16.62 ± 4.92 years in the PDR subgroup (p<0.001 in No-DR vs. NPDR, p<0.001 in No-DR vs. PDR, and p=0.144 in NPDR vs. PDR). Demographic and clinical characteristics of the DM subgroups are summarized in table 2.

**Table 1:** Demographic characteristics of the DM and control groups

	DM group (n=155)	Control group (n=145)	p value^*^
Age (years), mean ± SD (range)	55.2 ± 8.9 (26-73)	55.6 ± 7.2 (36-70)	0.605
Gender (male/female)	85/70	80/65	0.954

DM= diabetes mellitus; SD= standard deviation; M= male; F= female.

* Student’s t-test was used for age, and chi-square test was used for gender.

**Table 2:** Demographic and clinical characteristics of the No-DR, NPDR, and PDR groups

	No-DR group (n=49) Mean ± SD (range)	NPDR group (n=53) Mean ± SD (range)	PDR group (n=53) Mean ± SD (range)	p value^*^
Age (years)	54.3 ± 10.1 (26-73)	56.2 ± 7.4 (28-70)	55.0 ± 9.2 (27-71)	0.556
Gender (M/F)	27/22	29/24	29/24	0.999
DM duration (years)	8.3 ± 4.0 (3-20)	14.1 ± 4.8 (5-26)	16.6 ± 4.9 (8-30)	**<0.001** ^a^
HbA1c (%)	9.1 ± 2.5 (5.5-15.8)	9.5 ± 1.7 (6.0-13.4)	9.5 ± 2.2 (6.6-16.3)	0.553

DR= diabetic retinopathy; NPDR= nonproliferative diabetic retinopathy; PDR= proliferative diabetic retinopathy; SD= standard deviation; M= male; F= female; DM= diabetes mellitus.

^*^Student’s t-test was used for age and chi-square test was used for gender.

a= p<0.001 in No-DR vs. NPDR, p<0.001 in No-DR vs. PDR, and p=0.144 in NPDR vs. PDR

Upon analyzing the pupil diameter in static and dynamic pupillometry, there were statistically significant differences found between the DM and control groups (p<0.05 for all), as summarized in table 3.

**Table 3:** The results of pupil diameter in DM and control groups

			DM group (n=155) Mean ± SD (range)	Control group (n=145) Mean ± SD (range)	p value^*^	
Static pupillometry		Scotopic (mm)	4.2 ± 0.8 (2.3-6.4)	4.9 ± 0.7 (3.4-6.9)	**<0.001**	
		Mesopic (mm)	3.9 ± 0.7 (2.3-5.6)	4.4 ± 0.7 (2.5-6.3)	**<0.001**	
		Photopic (mm)	3.3 ± 0.6 (2.2-4.7)	3.5 ± 0.6 (2.4-5.5)	**0.007**	
	Dynamic pupillometry	0^th^ second (mm)	3.1 ± 0.6 (2.0-4.5)	3.3 ± 0.5 (2.3-5.0)	**0.005**	
		1^st^ second (mm)	3.6 ± 0.6 (2.3-5.1)	4.0 ± 0.6 (2.7-5.6)	**<0.001**	
		2^nd^ second (mm)	3.8 ± 0.7 (2.4-5.4)	4.3 ± 0.6 (3.0-5.9)	**<0.001**	
		4^th^ second (mm)	4.0 ± 0.8 (2.4-5.6)	4.6 ± 0.6 (3.0-6.3)	**<0.001**	
		6^th^ second (mm)	4.1 ± 0.8 (2.5-6.0)	4.8 ± 0.6 (3.3-6.6)	**<0.001**	
		8^th^ second (mm)	4.2 ± 0.8 (2.5-6.3)	4.9 ± 0.7 (3.4-6.7)	**<0.001**	
		10^th^ second (mm)	4.3 ± 0.8 (2.5-6.3)	4.9 ± 0.7 (3.5-6.8)	**<0.001**	

DM= diabetes mellitus; SD= standard deviation.

^*^Student’s t-test was used.

The DM subgroup analysis revealed statistically significant differences between the No-DR, NPDR, and PDR subgroups (p<0.001 for all). Pupil diameter results from static and dynamic pupillometry of the DM subgroups are summarized in table 4. As per the findings of static pupillometry under the scotopic condition, the No-DR, NPDR, and PDR subgroups were statistically different from one another (p=0.014 in No-DR vs. NPDR, p<0.001 in No-DR vs. PDR, and p<0.001 in NPDR vs. PDR). However, the results of dynamic pupillometry and static pupillometry in the mesopic and photopic conditions showed otherwise: findings for the No-DR and NPDR subgroups were similar regarding these measurements (p>0.05 for all), while those of the PDR subgroup were statistically significantly different from either (p<0.05 for all).

**Table 4:** The results of pupil diameter in No-DR, NPDR, and PDR groups

			No-DR group (n=49) Mean ± SD (range)	NPDR group (n=53) Mean ± SD (range)	PDR group (n=53) Mean ± SD (range)	p value^*^	
Static pupillometry		Scotopic (mm)	4.7 ± 0.7 (3.4-6.4)	4.3 ± 0.6 (3.0-5.9)	3.6 ± 0.8 (2.3-5.9)	**<0.001** ^a^	
		Mesopic (mm)	4.2 ± 0.7 (3.0-5.6)	4.0 ± 0.6 (3.0-5.6)	3.4 ± 0.7 (2.3-5.4)	**<0.001** ^b^	
		Photopic (mm)	3.4 ± 0.6 (2.5-4.7)	3.4 ± 0.7 (2.5-4.7)	3.0 ± 0.6 (2.2-4.3)	**<0.001** ^c^	
	Dynamic pupillometry	0^th^ second (mm)	3.2 ± 0.5 (2.2-4.5)	3.3 ± 0.5 (2.5-4.4)	2.9 ± 0.5 (2.0-4.1)	**<0.001** ^d^	
		1^st^ second (mm)	3.8 ± 0.6 (2.8-5.1)	3.8 ± 0.5 (2.9-5.0)	3.2 ± 0.6 (2.3-4.8)	**<0.001** ^e^	
		2^nd^ second (mm)	4.0 ± 0.6 (2.9-5.2)	4.0 ± 0.6 (3.1-5.4)	3.3 ± 0.6 (2.4-5.2)	**<0.001** ^f^	
		4^th^ second (mm)	4.3 ± 0.6 (3.1-5.6)	4.2 ± 0.6 (3.2-5.6)	3.4 ± 0.7 (2.4-5.5)	**<0.001** ^g^	
		6^th^ second (mm)	4.5 ± 0.7 (3.1-6.0)	4.3 ± 0.6 (3.3-5.9)	3.5 ± 0.7 (2.5-5.7)	**<0.001** ^h^	
		8^th^ second (mm)	4.6 ± 0.7 (3.2-6.3)	4.4 ± 0.7 (3.4-6.0)	3.6 ± 0.8 (2.5-5.7)	**<0.001** ^i^	
		10^th^ second (mm)	4.7 ± 0.7 (3.2-6.3)	4.5 ± 0.6 (3.5-6.1)	3.6 ± 0.8 (2.5-5.8)	**<0.001** ^j^	

DR= diabetic retinopathy; NPDR= nonproliferative diabetic retinopathy; PDR= proliferative diabetic retinopathy; SD= standard deviation^*^One-way analysis of variance was useda:

p=0.014 in No-DR vs. NPDR, p<0.001 in No-DR vs. PDR, and p<0.001 in NPDR vs. PDR.^**^ b: p=0.232 in No-DR vs. NPDR, p<0.001 in No-DR vs. PDR, and p<0.001 in NPDR vs. PDR.^**^ c: p=0.800 in No-DR vs. NPDR, p=0.015 in No-DR vs. PDR, and p=0.002 in NPDR vs. PDR.^**^ d: p=0.773 in No-DR vs. NPDR, p=0.005 in No-DR vs. PDR, and p<0.001 in NPDR vs. PDR.^**^ e: p=0.936 in No-DR vs. NPDR, p<0.001 in No-DR vs. PDR, and p<0.001 in NPDR vs. PDR.^**^ f: p=0.790 in No-DR vs. NPDR, p<0.001 in No-DR vs. PDR, and p<0.001 in NPDR vs. PDR.^**^ g: p=0.537 in No-DR vs. NPDR, p<0.001 in No-DR vs. PDR, and p<0.001 in NPDR vs. PDR.^**^ h: p=0.467 in No-DR vs. NPDR, p<0.001 in No-DR vs. PDR, and p<0.001 in NPDR vs. PDR.^**^ i: p=0.386 in No-DR vs. NPDR, p<0.001 in No-DR vs. PDR, and p<0.001 in NPDR vs. PDR.^**^ j: p=0.323 in No-DR vs. NPDR, p<0.001 in No-DR vs. PDR, and p<0.001 in NPDR vs. PDR.^**^

** Tukey post hoc test was used.

**Figure 1:**
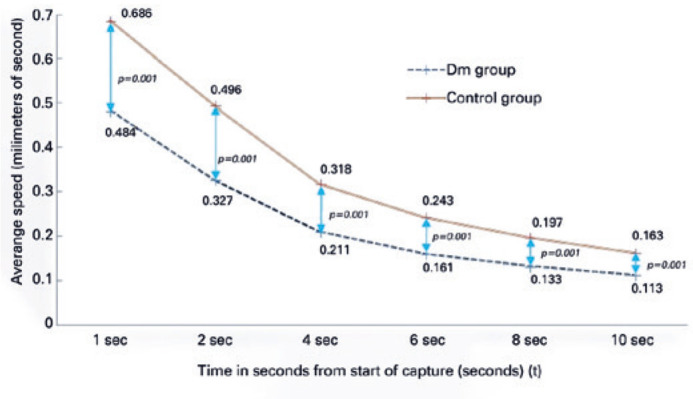
Demonstration of the average speeds of the DM and control groups.

**Figure 2:**
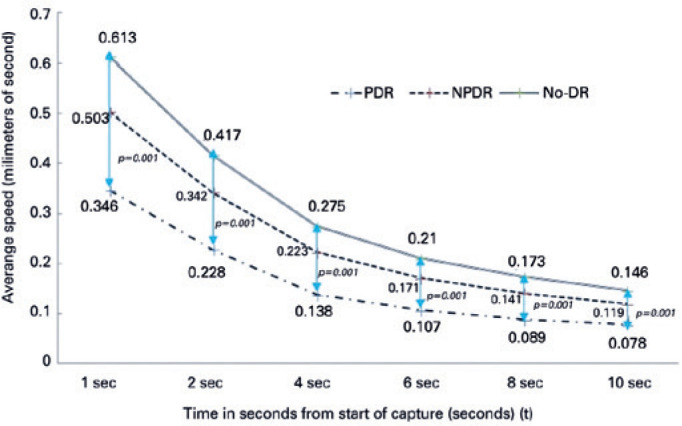
Demonstration of the average speeds of the diabetic subgroups

The average speed of pupillary dilation, another important parameter of dynamic pupillometry, was also measured. Of note, differences between the DM and control groups (p<0.001 for all) were statistically significant, as demonstrated in figure 1. Among the DM subgroups, the results of the PDR subgroup were significantly different, while those of the No-DR and NPDR subgroups were similar; these are summarized in table 5 and figure 2.

**Table 5:** Average speed of pupillary dilation in No-DR, NPDR, and PDR groups

	No-DR group (n=49) Mean ± SD (range)	NPDR group (n=53) Mean ± SD (range)	PDR group (n=53) Mean ± SD (range)	p value^*^
1^st^ second (mm/s)	0.6 ± 0.2 (0.4-0.8)	0.5 ± 0.2 (0.4-0.7)	0.4 ± 0.2 (0.3-0.5)	<0.001a
2^nd^ second (mm/s)	0.4 ± 0.2 (0.3-0.6)	0.3 ± 0.1 (0.2-0.4)	0.2 ± 0.1 (0.2-0.3)	<0.001b
4^th^ second (mm/s)	0.3 ± 0.2 (0.2-0.4)	0.2 ± 0.1 (0.2-0.3)	0.1 ± 0.1 (0.1-0.2)	<0.001c
6^th^ second (mm/s)	0.2 ± 0.1 (0.1-0.3)	0.2 ± 0.1 (0.1-0.3)	0.1 ± 0.1 (0.1-0.2)	<0.001d
8^th^ second (mm/s)	0.2 ± 0.1 (0.1-0.2)	0.1 ± 0.1 (0.1-0.2)	0.1 ± 0.1 (0.1-0.1)	<0.001e
10^th^ second (mm/s)	0.2 ± 0.1 (0.1-0.2)	0.1 ± 0.1 (0.1-0.2)	0.1 ± 0.1 (0.1-0.1)	<0.001f

DR= diabetic retinopathy; NPDR= nonproliferative diabetic retinopathy; PDR= proliferative diabetic retinopathy; SD= standard deviation^*^One-way analysis of variance was useda:

p=0.334 in No-DR vs. NPDR, p<0.001 in No-DR vs. PDR, and p <0.001 in NPDR vs. PDR.^**^ b: p=0.298 in No-DR vs. NPDR, p<0.001 in No-DR vs. PDR, and p<0.001 in NPDR vs. PDR.^**^ c: p=0.246 in No-DR vs. NPDR, p<0.001 in No-DR vs. PDR, and p<0.001 in NPDR vs. PDR.^**^ d: p=0.202 in No-DR vs. NPDR, p<0.001 in No-DR vs. PDR, and p<0.001 in NPDR vs. PDR.^**^ e: p=0.182 in No-DR vs. NPDR, p<0.001 in No-DR vs. PDR, and p<0.001 in NPDR vs. PDR.^**^ f: p=0.190 in No-DR vs. NPDR, p<0.001 in No-DR vs. PDR, and p<0.001 in NPDR vs. PDR.^**^

** Tukey post hoc test was used.

In table 6, correlations between static and dynamic pupil diameters were presented, taking into consideration the duration of DM and HbA1c levels. There were weak to moderate significant correlations between all pupil diameters in static and dynamic pupillometry with the duration of DM (p<0.05 for all). On the other hand, HbA1c values showed no statistically significant correlations with any of the investigated static and dynamic pupil diameters (p>0.05 for all).

**Table 6:** The correlations between pupil diameter with DM duration and HbA1c level

		DM duration (years)		HbA1c (%)	
		r value	p value^*^	r value	p value^*^
Static pupillometry	Scotopic (mm)	-0.480	**<0.001**	-0.100 0.214
	Mesopic (mm)	-0.375	**<0.001**	-0.079	0.328
	Photopic (mm)	-0.191	**<0.001**	-0.052	0.524
	Dynamic pupillometry	0^th^ second (mm)	-0.212	**<0.001**	-0.086	0.410	
		1^st^ second (mm)	-0.377	**<0.001**	-0.074	0.359	
		2^nd^ second (mm)	-0.446	**<0.001**	-0.079	0.329	
		4^th^ second (mm)	-0.487	**<0.001**	-0.074	0.360	
		6^th^ second (mm)	-0.507	**<0.001**	-0.085	0.293	
		8^th^ second (mm)	-0.504	**<0.001**	-0.083	0.302	
		10^th^ second (mm)	-0.502	**<0.001**	-0.077	0.341	

DM= diabetes mellitus.

^*^Pearson correlation coefficient test was used.

## DISCUSSION

Resting pupil size is mainly controlled by the sympathetic nervous system, and a decrease in resting pupil diameter is considered as a result of diminishing sympathetic outflow to the pupillary dilatator muscle^(12)^. In the pupillary construction phase, changes in pupil diameter and the duration of pupil size change are related to the parasympathetic nervous system. Separately, in the postconstruction recovery phase, the sympathetic and parasympathetic nervous systems work in harmony with each other^(13)^. Ferrari et al.^(14)^ stated that DM subjects have both sympathetic and parasympathetic dysfunctions, as evidenced by diminished amplitude reflexes and smaller pupil diameters. This study showed there are significant differences between DM and non-DM subjects in terms of pupil diameter in static and dynamic pupillometry and the average speed of pupillary dilation.

Some previous studies have suggested that pupillary parameters are altered in various groups of patients with DR. There is a very limited number of studies in literature in which DM subjects were grouped according to DR stages. Park et al.^(11)^ studied the pupillary functions of 50 DM subjects who did not have DR or NPDR in several stages and 25 healthy control subjects. They stated that the mean baseline pupil diameters of all NPDR groups in the dark were smaller than that in the control group. Additionally, the moderate-severe NPDR group was separated from the no NPDR and mild NPDR groups according to many other parameters^(11)^. Jain et al.^(15)^ studied cases containing either no DR, mild NPDR, moderate NPDR, severe NPDR, or PDR. They concluded that pupillary dynamics are abnormal in the early stages of DR and progress with increasing DR severity. They further investigated pupillary function with another technique; however, their study included both eyes of subjects, and they did not exclude type 1 DM^(15)^. Meanwhile in this study, patients were divided into categories No-DR, NPDR, and PDR. Firstly, it showed that pupil diameter is altered in patients with DM. Second, the pupillometry measurements are similar in DM patients without DR and with NPDR. In other words, the presence of NPDR does not provoke a significant difference in pupillometry measurements according to this study. Also, pupillometry measurements are more altered in DM patients with PDR. The most important finding of this study is that the characteristics of PDR differ significantly from other stages of DR.

Ortube et al.^(16)^ showed a statistically significant alteration in constriction velocity of moderate to severe NPDR cases when compared with a control group. According to their report, these values were highly correlated with the severity of the DR but not with the duration of the DM^(16)^. Interestingly, our study showed different results from this previous study. In our study, a weak to moderate significant relationship was found between all investigated pupil diameters with the duration of DM. This difference can be explained by the use of infrared pupillometry and the small subject group size. In addition, a relationship between pupillary function and DM duration was also determined. DR is a microangiopathy involving hypoxia in neuronal cells and the main pathophysiological mechanism of DM-related neuropathy^(17)^. The duration of DM is related to increased nerve fiber influences and changes in pupillary functions. With the extension of the duration of DM, more and more nerve fibers are affected, and pupillary functions are increasingly altered. Similar results were found by Cahill et al.^(18)^ with infrared pupillometry.

In pediatric patients with DM, Karavanaki et al.^(19)^ studied pupillary adaptation to darkness using a portable pupillometer and found that the mean pupil size was negatively correlated with HbA1c level. In contrast to their study, we did not find any correlations between HbA1c values and pupil diameter in static and dynamic pupillometry. However, there are many methodological differences between the two studies, with the most important one being the categorization of numerical values. Karavanaki et al.^(19)^ separated the DM patients as having either poor-, moderate-, or good-controlled HbA1c, and this differed from the uniformly high HbA1c levels observed in the present study.

Pittasch et al.^(4)^ and Cahill et al.^(18)^ in their respective investigations evaluated pupillary function using pharmacological manipulations. Ferrari et al.^(14)^ used a pupil stimulator and response recorder that document pupillary responses with a video camera after stimulating with white bright and infrared LEDs. Similarly, Park et al.^(11)^, Jain et al.^(15)^, and Yang et al.^(20)^ employed a pupillography system including an infrared-sensitive video camera and a luminometer. Prakash et al.^(10)^ measured pupil responses in normal subjects using a modern Scheimpflug-based automatic pupillometry system. This device could measure pupillary responses via either scotopic, mesopic, or photopic static pupillometry or dynamic pupillometry, yielding information about the behavior of the pupil under decreasing illumination conditions. Here, we measured pupil responses in DM subjects. Therefore, to our knowledge, our study is the first study that evaluated the pupillary function of DM patients by Scheimpflug-based automated pupillometry. Also, our study contains one of the largest sample sizes for this subject to date in literature (specifically, 300 subjects, with 155 having type 2 DM without or with DR in several stages).

We applied great care on elucidating the differences between the types of DM in our patients and evaluated patients with type 2 DM. Separating DM subjects is vital in the study because we know different pupillary responses can be observed in patients with type 1 versus type 2 DM^(18)^. In addition, the most important part of our study involved subjects with PDR. We included subjects with PDR from among newly diagnosed, previously untreated patients to exclude any effects of laser treatment on pupil responses, because it has been shown that panretinal laser photocoagulation may significantly affect pupil diameter; however, focal/grid laser photocoagulation may not^(21)^. Park et al.^(11)^ in their research did not include patients who had undergone panretinal laser photocoagulation, and we designed our study with reference to their method. Thus, we excluded the effects of generalized retinal cell death on pupillary function. In this regard, our study includes a homogeneous DM group as well as a large sample size.

The main goal of this study was to extensively investigate pupillary function in patients with type 2 DM, and it can be deemed different from the previous studies in terms of its design, methods, and results; nevertheless, this study also has several limitations. Systemic diseases, use of insulin or oral antidiabetics, and previous ocular treatments may affect pupillary measurements in DM patients, and it is utopian to think completely excluding these factors. Additionally, ultrastructure abnormalities in iris specimens, including sphincter and dilatator pupil muscle nerve endings, were observed in DM patients, but we did not study how these might affect pupillometry measurements^(22)^. These are possible topics that should be delved into in future research.

In conclusion, this study shows that static and dynamic pupillometry measurements are altered in patients with type 2 DM and that this alteration progresses as the duration of DM increases. The presence of NPDR does not have a negative effect on pupillometry findings, but it is more altered in the co-presence of PDR. These results suggest that automated quantitative pupillometry may be useful to verify the severity of DR.
